# Notch1 Is Involved in Physiologic Cardiac Hypertrophy of Mice via the p38 Signaling Pathway after Voluntary Running

**DOI:** 10.3390/ijms24043212

**Published:** 2023-02-06

**Authors:** Weiwei Zhang, Jiayi Liu, Zekang Wu, Guanwei Fan, Zhuo Yang, Chunhua Liu

**Affiliations:** 1 School of Medicine, Nankai University, Tianjin 300071, China; 2 State Key Laboratory of Modern Chinese Medicine, Tianjin University of Traditional Chinese Medicine, Tianjin 301617, China

**Keywords:** cardiac hypertrophy, voluntary running, Notch1, p38, autophagy

## Abstract

Appropriate exercise such as voluntary wheel-running can induce physiological cardiac hypertrophy. Notch1 plays an important role in cardiac hypertrophy; however, the experimental results are inconsistent. In this experiment, we aimed to explore the role of Notch1 in physiological cardiac hypertrophy. Twenty-nine adult male mice were randomly divided into a Notch1 heterozygous deficient control (Notch1^+/−^ CON) group, a Notch1 heterozygous deficient running (Notch1^+/−^ RUN) group, a wild type control (WT CON) group, and a wild type running (WT RUN) group. Mice in the Notch1^+/−^ RUN and WT RUN groups had access to voluntary wheel-running for two weeks. Next, the cardiac function of all of the mice was examined by echocardiography. The H&E staining, Masson trichrome staining, and a Western blot assay were carried out to analyze cardiac hypertrophy, cardiac fibrosis, and the expression of proteins relating to cardiac hypertrophy. After two-weeks of running, the Notch1 receptor expression was decreased in the hearts of the WT RUN group. The degree of cardiac hypertrophy in the Notch1^+/−^ RUN mice was lower than that of their littermate control. Compared to the Notch1^+/−^ CON group, Notch1 heterozygous deficiency could lead to a decrease in Beclin-1 expression and the ratio of LC3II/LC3I in the Notch1^+/−^ RUN group. The results suggest that Notch1 heterozygous deficiency could partly dampen the induction of autophagy. Moreover, Notch1 deficiency may lead to the inactivation of p38 and the reduction of β-catenin expression in the Notch1^+/−^ RUN group. In conclusion, Notch1 plays a critical role in physiologic cardiac hypertrophy through the p38 signaling pathway. Our results will help to understand the underlying mechanism of Notch1 on physiological cardiac hypertrophy.

## 1. Introduction

Appropriate exercise training can elicit an adaptive change in the heart, and this cardiac response is usually called physiological cardiac hypertrophy. Accumulating evidence has elucidated that activation of physiological hypertrophy can effectively reduce the risk of cardiovascular diseases and is conducive to the maintenance of normal physiological functions of the cardiovascular system [1,2]. As known, cardiac hypertrophy includes eccentric hypertrophy and concentric hypertrophy. Aerobic exercises, such as running and swimming, usually result in volume overload and eccentric hypertrophy [3]. Moreover, the cardiac function of physiological hypertrophy usually remains unchanged or enhanced, and the heart doesn’t have any functional or structural abnormity [4]. On the contrary, pathological cardiac hypertrophy is caused by pressure or volume overload, genetic mutations, and other diseases. It usually displays a high level of fetal gene expression, cardiac dysfunction, cardiac remodeling, and cardiac fibrosis. Finally, pathological cardiac hypertrophy often progresses toward heart failure, which could increase the mortality of patients [5].

Besides this, some important factors coordinate physiological and pathological cardiac hypertrophy. For example, the PI3K/Akt signaling pathway, p38 signaling pathway, and mTOR participate in the modulation of cardiac hypertrophy, all of which can affect gene transcription, protein translation, and metabolism. Mechanical stimulation can activate IGF1 and its downstream signal Akt, then the latter can induce myocardial hypertrophy [5]. Akt can inhibit the eukaryotic translation initiation factor 4E-binding protein 1(4E-BP1) and upregulate the CBP/p300- interacting transactivator with ED-richcarboxy-terminal domain 4 (CITED4) to enhance cardiac hypertrophy [6,7]. Moreover, the activation of Akt can inhibit GSK3β (GSK3β is an inhibitor of myocardial hypertrophy), and it will activate the expression of other genes related to myocardial hypertrophy [8,9]. Recently, several studies found that upregulation of p-Akt initially enhances cardiac hypertrophy as well as maintains cardiac function [10,11]. However, constitutive activation of Akt results in pathological hypertrophy and the impairment of cardiac function [12,13]. The references above suggested that the physiological level of p-Akt is beneficial for the heart.

Numerous studies on animals have demonstrated the effect of treadmill training and swimming on cardiac hypertrophy. A previous study reported exercise-induced physiological hypertrophy. When mice were trained with a treadmill for six weeks (five days/week), Akt-mTOR signaling increased [14]. Konstandin’s group and Konhilas’s group have reported that the heart-weight to body-weight ratio (HW/BW) of wild-type mice was increased after voluntary wheel running [15,16]. Besides this, a significant increase in autophagy activity was found in the hearts of exercise rats [17]. As known, exercise can bidirectionally regulate autophagy [18,19]. Specifically, if cardiovascular diseases were caused by insufficient autophagy, exercise training was able to up-regulate autophagy [20,21]. If the cardiovascular disease were caused by excessive autophagy, exercise training was able to inhibit autophagy and dampen the cardiovascular disease course [22,23].

Notch1 has been reported to be associated with a wild range of biological processes in embryogenesis and directly participates in the entire process of cardiac development [24]. It is a critical determinant of cardiac stem cell proliferation and differentiation. Inhibition of Notch1 signaling results in life-threatening dilated cardiomyopathy [25]. Upregulation of Notch1 in the hypertrophic heart controls the adaptive response of the heart to stress; it not only restricts the extent of the hypertrophic response but also contributes to cell survival in cardiomyocytes [26]. Besides being directly involved in heart development, Notch signaling can also crosstalk with other signaling pathways to play its roles, such as the PI3K/Akt and p38 signaling pathways [27,28]. Our previous study found that Notch1 participates in the voluntary running-enhanced cognitive function of mice [29]. However, the role Notch1 plays in voluntary running is still poorly understood. In this experiment, we evaluated the effect of Notch1 in voluntary wheel-running-induced cardiac hypertrophy of mice and explored the relevant molecular mechanism of Notch1 in this process.

## 2. Results

### 2.1. Notch1 Deficiency Made No Difference in the Mice’s Running Ability

**Figure 1:**
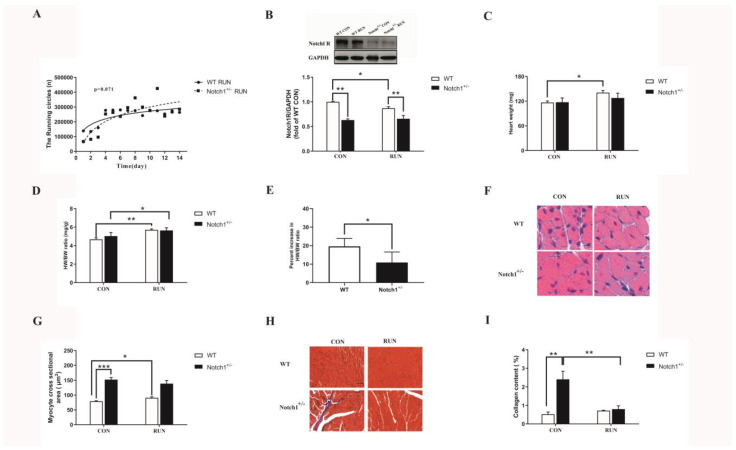
(**A**): The running circles of WT RUN group (n = 8) and Notch1^+/−^ RUN group (n = 6); (**B**): The Notch1 expression (n = 4); (**C**): Heart weight (mg) (n = 6–8); (**D**): HW/BW ratio (mg/g) (n = 6–8); (**E**): the percent increase in HW/BW ratio; (**F**): The representative optical microscope photographs of cardiomyocytes (scale bar = 10 μm); (**G**): the myocyte cross-sectional area (n = 4); (**H**): The representative photographs of Masson staining (scale bar = 20 μm); (**I**): the collagen content (n = 4). WT CON: wild-type control mice; WT RUN: wild-type running mice; Notch1^+/−^ CON: Notch1 heterozygote deficient control mice; Notch1^+/−^ RUN: Notch1 heterozygote deficient running mice. Data are expressed as mean ± S.E.M. * *p* < 0.05, ** *p* < 0.01, *** *p* < 0.001.

To avoid social isolation disturbance, the mice were raised in groups of four. As shown in Figure 1A, one dot presents the average distance of one mouse per day. There was no significant difference between the Notch1^+/−^ RUN and WT RUN groups (*p* > 0.05). That is to say, Notch1 heterozygous deficiency did not influence the running ability of the mice.

### 2.2. Notch1 Was Altered by Voluntary Running in the WT RUN Group

As shown in Figure 1B, the level of the Notch1 expression in the WT RUN group was lower than that of the WT CON group, which suggests that Notch1 was altered. On the contrary, Notch1 expressions in the Notch1^+/−^ CON and Notch1^+/−^ RUN groups were lower than that of their littermate controls (*p* < 0.01), which suggests that the Notch1 gene had been knocked -down. After voluntary running, there was no difference between the Notch1^+/−^ CON and Notch1^+/−^ RUN groups (*p* > 0.05). This suggests that voluntary running could not alter the Notch1 expression in these Notch1 heterozygous deficiency mice.

### 2.3. Notch1 Heterozygous Deficiency Partly Obstructed the Degree of Cardiomyocytes Hypertrophy after Voluntary Running

As shown in Figure 1C,D, the heart weight and the HW/BW ratio in the WT RUN group were higher than those of the WT CON group (*p* < 0.05, Figure 1C; *p* < 0.01, Figure 1D). This suggests that voluntary running did increase the HW/BW ratio. On the contrary, the HW/BW ratio in the Notch1^+/−^ RUN group was increased to 5.74 ± 0.29 compared to that in the WT RUN group, and no difference was found between these two groups (*p* > 0.05, Figure 1D). However, the percent increase in the HW/BW ratio was decreased in the Notch1^+/−^ mice compared to their WT littermates (*p* < 0.05, Figure 1E). Then, we observed the morphology of the hearts. As shown in Figure 1F,G, compared to the WT CON group, the cardiomyocyte cross-sectional area was increased in the WT RUN group, which suggests that voluntary running did induce cardiac hypertrophy in the wild-type mice. At the same time, the cross-sectional area of the cardiac cell in the Notch1^+/−^ CON mice was bigger than that of the WT CON group (*p* < 0.05, Figure 1F,G). After voluntary running, the myocyte cross-sectional area was not increased in the Notch1^+/−^ RUN mice compared to the Notch1^+/−^ CON mice (*p* > 0.05, Figure 1F,G). This implies that Notch1 may play an important role in the degree of cardiac hypertrophy after voluntary running. In addition, we also observed that there was lesser fibrosis in the Notch1^+/−^ RUN group than that in the Notch1^+/−^ CON group (*p* < 0.01, Figure 1H,I). This suggests that voluntary running may be beneficial to reduce the degree of fibrosis of mice in the Notch1^+/−^ RUN group.

**Figure 2:**
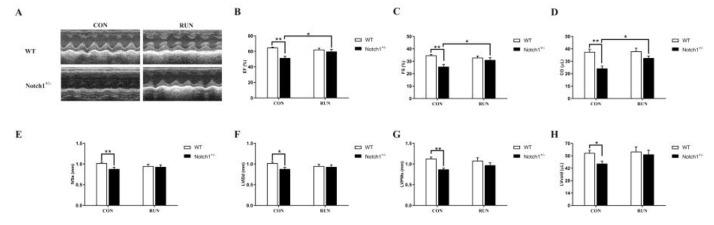
The cardiac function of all mice by echocardiography. (**A**): The representative pictures of echocardiography; (**B**): EF; (**C**): FS; (**D**): CO; (**E**): IVSs; (**F**): LVIDd; (**G**): LVPWs; (**H**): LVvold. IVSs: interventricular septum systolic thickness, LVIDd: left ventricular dimension in diastole, LVPWs: left ventricular posterior wall systolic thickness, EF: ejection fractional, FS: fractional shortening, LVvold: Left ventricular diastolic volume, CO: cardiac output per beat. WT CON: wild-type control mice; WT RUN: wild-type running mice; Notch1^+/−^ CON: Notch1 heterozygote deficient control mice; Notch1^+/−^ RUN: Notch1 heterozygote deficient running mice. Data are expressed as mean ± S.E.M. * *p* < 0.05, ** *p* < 0.01. n = 6–8 per group.

In this experiment, we also evaluate the mice’s cardiac function by echocardiography. Compared to the WT CON group, the values of LVIDd, EF, FS, LVvold, and CO were decreased in the Notch1^+/−^ CON group (*p* < 0.01, Figure 2A–H). It indicated that the cardiac function was reduced. After two weeks of voluntary running, the FS, EF, and CO were partly improved in the Notch1^+/−^ RUN group compared to the Notch1^+/−^ CON group (*p* < 0.05, Figure 2A–D). There was no significant difference in the IVSs, LVPWs, LVIDd, and LVvold between the Notch1^+/−^ RUN group and the Notch1^+/−^ CON group; at the same time, there was also no difference in the IVSs, LVPWs, LVIDd, and LVvold between the Notch1^+/−^ RUN and WT RUN groups, as well as between the Notch1^+/−^ RUN and WT CON group (*p* > 0.05, Figure 2E–H). This suggests that the cardiac function of the Notch1^+/−^ RUN group was partly improved after voluntary running.

### 2.4. Notch1 Heterozygous Deficiency May Partly Dampen the Activation of Autophagy and the p38 Signaling Pathway after Voluntary Running

**Figure 3:**
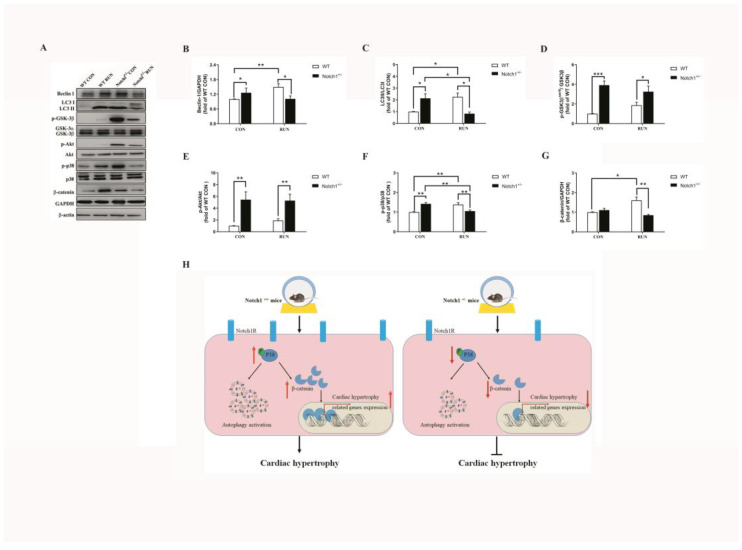
The expression of cardiac hypertrophy-associated protein detected by Western blot assay in the hearts of mice. (**A**): The representative Western blot bands; (**B**): Quantitative analysis of the Beclin-1 expression(n = 6); (**C**): Quantitative analysis of the LC3II/LC3I(n = 6); (**D**): Quantitative analysis of the p-GSK3β^ser9^ level (n = 6); (**E**): Quantitative analysis of the β-catenin expression (n = 6); (**F**): Quantitative analysis of the p-Akt level (n = 6); (**G**): Quantitative analysis of the p-p38 level (n = 6). (**H**). The speculated mechanism of Notch1 on mouse cardiac hypertrophy in response to voluntary running. 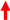: increase; 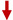: decrease; 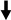: activate; 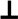: inhibit. WT CON: wild-type control mice; WT RUN: wild-type running mice; Notch1^+/−^ CON: Notch1 heterozygote deficient control mice; Notch1^+/−^ RUN: Notch1 heterozygote deficient running mice. Data are expressed as mean ± S.E.M. * *p* < 0.05, ** *p* < 0.01, *** *p* < 0.001.

As shown in Figure 3, compared with the WT CON group, the Beclin-1 expression and the ratio of LC3II/LC3I were increased in the WT RUN group (*p* < 0.05, Figure 3A–C). This suggests that autophagy was induced in the wild-type mice after voluntary running. However, no increase in the Beclin-1 expression was detected in the Notch1^+/−^ RUN group, and no difference was found compared to the Notch1^+/−^ CON and WT CON groups (*p* > 0.05, Figure 3A,B). This suggests that the Beclin-1 expression of the Notch1^+/−^ RUN group appeared to be unaffected by voluntary running. Further analysis showed that the ratio of LC3II/LC3I in the Notch1^+/−^ RUN group was reduced to 0.83 ± 0.14 (fold of the WT CON group) compared with the Notch1^+/−^ CON group (*p* < 0.05, Figure 3A,C) and no difference was found compared with the WT CON group (*p* > 0.05, Figure 3A,C). These results suggest that the induction of autophagy may be dampened in the Notch1^+/−^ RUN group after voluntary running.

Studies have reported that the Akt/GSK3β and p38 signaling pathways participate in cardiac hypertrophy [30,31]. In this experiment, we examined them. As shown in Figure 3, p-Akt^ser 473^/Akt and p-GSK3β^ser9^/GSK3β in the WT RUN group were increased, however, no difference was found between the WT RUN and WT CON groups (*p* > 0.05, Figure 3A,D,E). On the contrary, p-Akt^ser473^ and p-GSK3β^ser9^ of the Notch1^+/−^ mice were markedly increased compared to those of their littermate controls (*p* < 0.05, Figure 3D,E). Further analysis showed no difference in p-Akt^ser473^ and p-GSK3β^ser9^ between the Notch1^+/−^ CON and Notch1^+/−^ RUN groups (*p* > 0.05, Figure 3D,E). This implied that deficiency of Notch1 could not activate the Akt/GSK3β signaling pathway after two weeks of voluntary running. As shown in Figure 3A,F, the phosphorylation of p38 was increased to 1.38 ± 0.10 (fold of the WT CON group) in the WT RUN group (*p* < 0.01, Figure 3A,F). At the same time, the phosphorylation of p38 in the Notch1^+/−^ CON group was higher than that in the WT CON group (*p* < 0.01, Figure 3F). However, this trend disappeared in the Notch1^+/−^ RUN group. Compared to the Notch1^+/−^ CON group, the phosphorylation of p38 was decreased to 1.05 ± 0.06 (fold of the WT CON group) in the Notch1^+/−^ RUN group (*p* < 0.01, Figure 3A,F). Moreover, there was no difference between the Notch1^+/−^ RUN and WT CON groups (*p* > 0.05, Figure 3A,F). This suggests that the deficiency of Notch1 may disturb the activation of p38 after exercise.

It has been reported that exercise up-regulates the expression of β-catenin [32]. As shown in Figure 3, the WT RUN group had a higher level of β-catenin expression than the WT CON group (*p* < 0.05, Figure 3A,G). This suggested that voluntary running did increase the β-catenin expression. Moreover, the β-catenin expression was increased to 1.11 ± 0.09 (fold of the WT CON group) in the Notch1^+/−^ CON group, but no difference was detected between the WT CON and Notch1^+/−^ CON groups (*p* > 0.05, Figure 3A,G). On the contrary, the β-catenin expression of the Notch1^+/−^ RUN group was reduced to 0.85 ± 0.04 (fold of the WT CON group), and there was no significant difference between the Notch1^+/−^ RUN and Notch1^+/−^ CON groups (*p* > 0.05, Figure 3A,E). Interestingly, there was a difference between the Notch1^+/−^ RUN and WT RUN group. This suggests that the deficiency of Notch1 may decrease the β-catenin expression after exercise (Figure 3H).

## 3. Materials and Methods 

### 3.1. Animals

Studies have reported that ERβ is involved in the response of the heart to exercise. Therefore, to avoid sex-dependent effects, only males were included in this study. Because the homozygous null mutation of the Notch1 gene is embryonically lethal, we used mice carrying a heterozygous null mutation of the Notch1 gene in this experiment [33,34]. Adult male heterozygous mice (Notch1^+/−^) and wild type (WT) littermates (Notch1^+/+^ C57BL/6J) were provided by the Institute of Laboratory Animal Science, Chinese Academy of Medical Sciences, and all mice were 7–8 weeks of age. The genotypes of all mice were identified by the supplier. Twenty-nine mice were randomly divided into four different groups, i.e., a wild-type control group (WT CON) (n = 8), a wild-type running group (WT RUN) (n = 8), a Notch1^+/−^ control group (Notch1^+/−^ CON) (n = 7), and a Notch1^+/−^ running group (Notch1^+/−^ RUN) (n = 6). All mice were reared in a specific pathogen-free (SPF) house with a temperature and humidity-controlled facility (21–23 °C) and water were provided ad libitum. All mice were housed in groups of four. The investigation was approved by the Animal Research Ethics Committee, School of Medicine, Nankai University. All animal experiments were performed following the National Institute of Health Guide for the Care and Use of Laboratory Animals (NIH Publications No. 80–23) revised in 1996 and the Animal Management Rules of the Ministry of Health of the People’s Republic of China.

### 3.2. Experimental Design

The WT RUN and Notch1^+/−^ RUN mice had access to voluntary wheel-running for 2 weeks. The WT CON and Notch1^+/−^ CON mice were not subjected to voluntary wheel running. Two weeks later, all mice were anesthetized with 1.5% isoflurane after induction with isoflurane at 3% for 2 min, and echocardiography was carried out to assess the function of the heart [35]. After echocardiography, mice were sacrificed via 5% isoflurane inhalation [36] and the hearts were collected for the next experiments.

### 3.3. Voluntary Running

To avoid social isolation-induced stress, all mice were reared in groups of four [37,38,39]. Voluntary running was performed as previously described [29]. Briefly, mice in both the WT RUN and Notch1^+/−^ RUN groups were reared in cages with voluntary running wheels for 2 weeks, and the diameter of the running wheel was 13 cm. The running wheels were equipped with electronic counters to record the daily circles in each cage. Therefore, 14 samples per group were obtained. Meanwhile, mice in the WT CON and Notch1^+/−^ CON groups were reared in cages without running wheels.

### 3.4. Cardiac Function Assessment

On the 15th day, echocardiography was carried out using an ultra-high-resolution small animal ultrasound imaging system (Visual Sonics Vevo 2100, Toronto, ON, Canada). Interventricular septum diastolic thickness (IVSd), left ventricular dimension in diastole (LVIDd), left ventricular posterior wall diastolic thickness (LVPWd), interventricular septum systolic thickness (IVSs), left ventricular dimension in systole (LVIDs), left ventricular posterior wall systolic thickness (LVPWs), left ventricular ejection fraction (EF), left ventricular fractional shortening (FS), left ventricular end-diastolic volume (LVvold), left ventricular end-systolic volume (LVvols) and cardiac output per beat (CO) were detected. All data were analyzed with the software of the ultrasound system.

### 3.5. Hematoxylin-Eosin (H&E) Staining and Masson Trichrome Staining

After echocardiographic evaluation, all mice were immediately sacrificed. Hearts were collected, then put into PBS at 4 °C, and squeezed to remove the residual blood. Then, some hearts were frozen at −80 °C, while the others were immersed in 4% paraformaldehyde for 48 h, embedded in paraffin, cut into 5 μm cross-sections, and stained with hematoxylin-eosin (H&E) and Masson trichrome staining according to the manufacturer’s protocol. In the H&E staining experiment, left ventricular sections were photographed by light microscopy. The circular or oval cardiac cells sections were considered the suitable cross-sections. Then, the outline of each cardiac cell was traced and the mean area of each cell was calculated by Image J software. Fifteen to twenty cells per section were analyzed, and four sections were selected from each heart. The mean data reflected results from four hearts in each group. In the Masson trichrome staining experiment, fibrosis areas appeared blue and non-fibrotic areas appeared red. The fibrosis areas were also calculated by Image J software. The collagen content was expressed as a percentage of the total image area. In this experiment, five images were selected from each heart and the mean data reflected results from four hearts in each group.

### 3.6. Western Blot Assay

The Western blot assay was carried out as reported [40,41]. Briefly, the hearts were homogenized by protein lysis buffer at 4 °C. (Beyotime Biotechnology, Lianyungang, Jiangsu, China). Then, the supernatant was transferred to other tubes after being centrifuged at 12,000 rpm for 15 min at 4 °C. Thirty microgram proteins were separated by 10% or 13% SDS-PAGE gels and transferred onto the PVDF membrane (Promega Co., Ltd., Madison, WI, USA) at 4 °C. After 1 h blocking at room temperature, the membrane was incubated with primary antibodies for 12h at 4 °C, including Notch1 receptor (ab52627, 1:2000, Abcam, Cambridge, UK), Akt (#4691, 1:2000, CST, Boston, MA, USA), p-Akt^Ser473^ (#4060, 1:2000, CST, Boston, MA, USA), GSK3α/β (#5676, 1:2000, Abcam, Cambridge, UK), p-GSK3β^Ser9^ (#5558, 1:2000, Abcam, Cambridge, UK), Beclin1(ab207612, 1:2000, Abcam, Cambridge, UK), β-catenin (#8480, 1:2000, CST, Boston, MA, USA), p-p38^Thr180/Tyr182^(# 4511S, 1:2000, CST, Boston, MA, USA), p38 (# 9212S, 1:2000, CST, Boston, MA, USA), β-actin (#3700, 1:2000, CST, Boston, MA, USA), and GAPDH (#5174, 1:2000, CST, Boston, MA, USA). The mouse monoclonal antibody of LC3 was purchased from Medical & Biological Laboratories (M152-3, 1:1000, Tokyo, Japan). Finally, the membrane was incubated with secondary antibodies (W4011 and W4021, 1:5000, Promega, Madison, WI, USA). The protein band intensities were detected by Tanon 5500 chemiluminescent imaging system (Tanon Science and Technology, Shanghai, China).

### 3.7. Statistical Analysis

Data were shown as means ± S.E.M. The cardiac function and Western blot assay results were analyzed with two-way ANOVA. The least significant difference (LSD) test was used as the post-hoc test for the analysis of differences between the groups. The Mann-Whitney U-test was performed to compare the difference in Figure 3A. Statistical analysis was performed using SPSS 22.0 software. A probability value of *p* < 0.05 was considered significant. Pictures were processed with Photoshop software.

## 4. Discussion

Voluntary wheel running is considered an appropriate model to investigate the influences of exercise in mice. Compare to other exercise forms, the running pattern of voluntary wheel running is carried out under non-stressed conditions and is much more similar to the natural running behavior of mice [42]. Therefore, we selected the voluntary wheel running in this experiment. Comparing the running distance between these two RUN groups, there was no significant difference in running distance. This shows that Notch1 deficiency did not affect the exercise ability of the mice, which was similar to our previous study [29]. In addition, our data showed that the expression of Notch1 was decreased in the WT RUN group after two weeks of voluntary running. Our data suggested that Notch 1 was involved in voluntary running. When Notch1 was deficient, voluntary running could not alter the Notch1 expression in the Notch1^+/−^ RUN group. 

Physiological cardiac hypertrophy is usually presented as the normal organization of cardiac structure and normal/enhanced cardiac function. In addition, physiological hypertrophy is usually reversible [5]. As known, aerobic exercise, such as voluntary running, can induce physiological cardiac hypertrophy. Moreover, aerobic exercise usually leads to volume overload and eccentric hypertrophy [3]. In eccentric hypertrophy, the relative wall thickness may be normal, decreased, or increased [43]. On the contrary, pathological cardiac hypertrophy commonly presents a high level of fetal genes, fibrosis, remodeling, and cardiac dysfunction. Moreover, pathological hypertrophy is usually unreversible [5]. Our data showed that there was no significant difference between the cardiac function of the WT RUN and WT CON groups in this experiment, which is consistent with Hydoc’s results [44]. However, a deficiency of Notch1 led to a decrease in cardiac function. It has been reported that inhibition of Notch1-dependent cardiomyogenesis could lead to a dilated myopathy in the neonatal heart [25]. Based on these references, we consider that the discrepancy between the echocardiographic and the histological data in Notch1^+/−^ CON group may be due to eccentric cardiac hypertrophy. In addition, the reason for the decrease in cardiac function in the Notch1^+/−^ mice might be that Notch1 participated in the regulation of cardiovascular generation and proliferation, differentiation, and migration of cardiac stem cells [26]. After exercise, cardiac function was partly improved. Moreover, there was also no significant difference in cardiac function between the WT RUN and Notch1^+/−^ RUN groups. 

Our data showed that the heart weight and the HW/BW ratio of the mice were increased in the WT RUN group, which is consistent with Konhilas’s and Konstandin’s results [15,16]. Further analysis found that the percent increase of the HW/BW ratio in the Notch1^+/−^ mice was lower than that of its littermate controls. Combined with the morphological staining data, our results suggest that the deficiency of Notch1 led to a lower degree of cardiac hypertrophy after voluntary running. Considering the running duration of mice, we were not sure whether or not there would be a difference in the degree of cardiac hypertrophy between the Notch1^+/−^ CON and Notch1^+/−^ RUN groups after running longer.

How did Notch1 participate in cardiac hypertrophy? Mechanical stimulation could activate Akt/GSK3β, and then promote myocardial hypertrophy [45]. Moreover, p-Akt upregulated CITED4 to promote myocardial hypertrophy by promoting the expression of GATA4 [5]. β-catenin was able to transfer into the nucleus to affect the expression of hypertrophy-related genes [46]. When β-catenin haplo-insufficient mice were subjected to endurance training, the cardiomyocytes’ hypertrophic growth was attenuated [47]. This suggests that inhibition of β-catenin could decrease cardiac hypertrophy. GSK3β could phosphorylate β-catenin, thus inactivating it by ubiquitination and inhibiting the expression of genes related to myocardial hypertrophy [48,49]. On the contrary, p-Akt could phosphorylate GSK3β at ser 9 to make it inactive. Then, the inactive GSK3β could promote β-catenin to enter the nucleus and cause cardiac hypertrophy [50]. In this experiment, the Akt/GSK-3β pathway was not activated in the WT RUN mice, which is consistent with Elke’s results [51]. When Notch1 was deficient, the phosphorylation levels of Akt and GSK-3β were abnormally shot up in the Notch1^+/−^ mice, and no differences were found between the Notch1^+/−^ CON and Notch1^+/−^ RUN groups. These results infer that the Akt/GSK-3β pathway was also not activated when the Notch1^+/−^ mice were subjected to voluntary running. According to Chulan Kwon’s study, Notch1 was able to physically interact with unphosphorylated (active) β-catenin in stem and colon cancer cells, and negatively regulate the post-translational accumulation of active β-catenin protein. Interestingly, this modulation did not require the cleavage of Notch1 or GSK-3β-dependent activity of the β-catenin destruction complex [30]. In the WT RUN group, the Notch1 receptor expression was lower; conversely, the level of β-catenin was higher than that of the WT CON group. We speculate that Notch1 may directly modulate the β-catenin via physical interaction. As for the Notch1^+/−^ mice, there was a slight increase in β-catenin expression in the Notch1^+/−^ CON group, but there was no difference between the WT CON and Notch1^+/−^ CON groups. High levels of p-Akt/Akt and p-GSK-3β did not lead to a high level of β-catenin in the Notch1^+/−^ CON group. On the contrary, the β-catenin expression of the Notch1^+/−^ RUN group was decreased. Therefore, we posit that there may be another way to modulate the expression of β-catenin in Notch1^+/−^ mice.

p38 is another important signaling pathway, and it also participates in the hypertrophy reaction of myocardial cells [52]. As known, there are four subtypes in the p38 family, that is p38 alpha, p38 beta, p38 gamma, and p38 delta. p38 beta activation in cultured cardiomyocytes induces characteristic features of hypertrophy [53], whereas p38 alpha activation promotes cardiomyocyte apoptosis [54]. Moreover, the study also reported that the localization differences would result in access to different substrates and hence distinct functional effects [55]. These references suggested that different subtypes of p38 present different roles in cardiac hypertrophy. After exercise, the protein content of phosphorylated p38 was increased [52]. Inhibition of p38 protected hearts from cardiac hypertrophy [56,57]. In addition, the loss of p38 function reduced β-catenin nuclear accumulation in primary vascular smooth muscle cells (VSMCs) [31]. Conversely, active p38 signaling increases β-catenin nuclear localization and targeted gene activity in multiple cell types [31]. This implied that the p38 signaling pathway was able to regulate cardiac hypertrophy via β-catenin. In this experiment, the expression of p38 and β-catenin of the WT RUN group was higher than that of the Notch1^+/−^ RUN and WT CON groups. We infer that a deficiency of Notch1 may affect the activation of p38 after voluntary running, then it could reduce the β-catenin expression, and finally decrease the degree of cardiac hypertrophy.

As known, autophagy is important for cell homeostasis, and it is dependent on the lysosomal degradation pathway [58,59]. When autophagy occurs, autophagosomes can swallow misfolded protein, as well as impaired organelles. Next, all of them are transported into the lysosomes for degradation [60]. The homeostasis of autophagy (basal autophagy) plays a key role in cellular function and the development of physiological and pathological conditions [60]. Many studies have explored that exercise can induce autophagy in a short period [20,61,62]. At the same time, Rocchi and He found that both forced treadmill exercise and voluntary wheel running can induce autophagy in vivo [63]. This hints a close correlation between exercise and autophagy. In our experiment, we found that cardiac autophagy was increased in the WT RUN group, which is consistent with Rocchi’s results [63]. At the same time, the cardiac autophagy of the Notch1^+/−^ CON group was also increased, accompanied by cardiac hypertrophy and a decrease in cardiac function. We infer that the increased autophagy of Notch1^+/−^ CON may be a response to Notch1 knocked-down. After voluntary running, the cardiac autophagy of the Notch1^+/−^ RUN group was decreased, accompanied by improved cardiac function. This suggests that Notch1 may participate in the activation of cardiac autophagy. In addition, studies have reported that there is also a relationship between p38 and autophagy. Lin et al. reported that inhibition of p38MAPK attenuated not only mechanical stretch-induced cardiomyocyte hypertrophy but also autophagy [64]. Similarly, Wang et al. found that nicotine-induced cardiomyocytes hypertrophy through activation of autophagy accompanied by a high level of p-p38 [65]. Parr et al. revealed that inactivation (phosphorylation) of glycogen synthase kinase-3(GSK3) led to autophagy enhancement [66,67]. Moreover, the SB203080 (a specific p38 inhibitor) reduced the p-GSK3β^ser9^ and autophagy induced by MG132 in MDA-MB-231 cells [68]. In this experiment, the p38 signaling pathway was activated, at the same time, the autophagy was increased in the WT RUN and Notch1^+/−^ CON group. As to the Notch1^+/−^ RUN group, there were high levels of p-Akt and p-GSK3β^ser9^ compared to that of the WT RUN group, but the p38 signaling pathway was not activated. We speculated that the lower level of autophagy in the Notch1^+/−^ RUN group may be related to the inactivation of the p38 signaling pathway (Figure 3H).

In summary, we found that Notch1 may be involved in the cardiac hypertrophy process of mice caused by voluntary running. Deficiency of Notch1 could reduce the degree of cardiac hypertrophy. Interestingly, Notch1 deficiency led to the inactivation of p38, which could reduce the β-catenin expression and inhibit autophagy in the hearts of the mice. We posit that our study will provide a potential therapy for cardiac hypertrophy, especially for patients, whose heart disease is related to the Notch1 gene. However, there is still a limitation to our study. Given the genetic background of mice, the Notch1 gene is lacking in some organs and cells of mice (such as the heart, brain, kidney, lung, liver, T cells, and vascular endothelial cells), so we can not completely exclude the influence of Notch1 on development. Therefore, further studies are needed to investigate cardiac-specific knocked-out Notch1 mice in the future.

## Data Availability

The data that support the findings of this study are available on request from the corresponding author (C.L.) upon reasonable request.
